# Revisiting Social Value Orientations and Environmental Attitude–Identity–Intention in Decomposed Games

**DOI:** 10.3390/ijerph19126961

**Published:** 2022-06-07

**Authors:** Daniel Curtin, Fanli Jia

**Affiliations:** 1 Department of Psychology, Seton Hall University, South Orange, NJ 07828, USA; danielcurtin85@gmail.com; 2 Busch School of Business, The Catholic University of American, Washington, DC 20064, USA

**Keywords:** social value orientation, decomposed game, environmentalism, prosocial, proself, attitude, identity, behavior

## Abstract

Past research has identified social value orientation (e.g., prosocial vs. proself) as possible underlying facilitators of pro-environmental intentions. However, recent studies have failed to draw a causal relationship using an experimental design such as priming. The current study attempted to address this issue by revisiting the relationship using a decomposed game. In addition, the current study extended the relationship between social value orientation and different aspects of pro-environmentalism (e.g., environmental attitude, identity, and self-reported pro-environmental intention). The “Attitude–Identity–Intention” path was explored in prosocial and proself groups. One hundred and fifty participants completed the decomposed game (prosocial and proself value orientations) and their respective environmental attitude, identity, and self-reported pro-environmental intentions (PEIs) were compared. We found that prosocial participants had higher levels of environmental identity, attitude, and self-reported participatory PEIs than proself participants, but not on the leadership PEIs. In addition, environmental identity mediated the relationship between environmental attitude and self-reported PEIs. This mediation only existed among the prosocial participants. The results suggest that the decomposed game is still a valid measure in social value orientation and the relationship can be extended to different aspects of environmentalism.

## 1. Introduction

Research in the field of conservation/environmental psychology has been influenced by the study of social value orientation (SVO). Social value orientation is a person’s inclination to allocate resources between the self and another [1]. Three social value orientations were categorized in past research [2,3,4]: Firstly, the “prosocial” orientation is categorized as beneficial to both oneself and others; secondly, the “individualist” orientation maximizes resources for one’s own gain; and thirdly, the “competitive” orientation maximizes the relative gain between oneself and others. Recent works have labeled the individualist and competitive orientations as “proself” [4], compared to the prosocial orientation. Van Lange, Joireman, Parks and Dijk [5] have discussed the importance of SVOs and argued that they impact individual decision-making regarding environmental issues.

Previous research has established the relationship between social value orientation (e.g., prosocial orientation) and pro-environmental intention. Steg and Vlek [6] reviewed the literature in pro-environmental intention/behavior and argued that values beyond one’s own interests (such as prosocial, altruistic, and self-transcendent) influence people’s engagement in PEIs. Nolan and Schulz [7] further reviewed different theories (e.g., Norm-Activation; Value–Belief–Norm Extension; Empathy–Altruism; Bystander; and Negative-State Relief) and concluded that prosocial values can promote pro-environmental engagement. Kramer, McClintock, and Messick [8] found that, when compared with their individualistic or competitive counterparts, prosocial participants were significantly less likely to overharvest a finite resource. Kaiser and Byrka [9] found that their pro-environmentally labeled participants were significantly more prosocial than their anti-environmentally labeled participants. Neaman, Otto, and Vinokur [10] further demonstrated that prosocial and pro-environmental behaviors are the “two facets of a broader trait”, which, in turn, strengthen the environmental educational programs. Other studies also found a similar pattern between prosocial orientation and specific environmental decision-making such as commuting behaviors [11], environmental political behaviors [12], and daily participatory environmental actions [13].

Studies addressing social value orientation and pro-environmental intention have utilized different experimental procedures. One common method is priming. Fischer et al. [14] conducted two experimental studies to temporarily change value goals using multiple priming tasks, including a word search puzzle, a sentence scrambling task, an autobiographical recall, and a similarity–differences with family and friend task. They found no difference between the experimental condition and the control condition regarding participants’ behavioral choices. Similar, Curtin and Jia [13] primed participants using autobiographical memories, but they did not find the group differences on self-reported pro-environmental actions. These studies suggest that priming social value orientations cannot influence people’s conservational behaviors due to a methodological issue (e.g., priming).

Another common method is a “decomposed game” [1]. In the decomposed game, a resource conservation task is set up so that participants decide how many valuable points they take from a collective resource pool. As the participants take more points for themselves (proself), the collective resource pool is depleted until no points are left for others. Alternatively, the participants can take some points for themselves and leave a relative amount to others (prosocial). Van Lange and other colleagues [2,3,15] revised the tasks in a paper-pencil/computer version in order to categorize prosocial (maximizing joint gains) and proself (either maximizing individual gains or maximize the differences between own and others). This revised task has been validated and examined in relation to PEI (see Pletzer et al. [16] for an example of a meta-analysis using the measure).

Even though the relationship between social value orientation and pro-environmental intention have been established, there was very little research to examine other aspects of pro-environmentalism. Environmentalism is a multifaceted and broad construct partially defined as the behavioral tendency to take actions involving pro-environmental commitments [17]. Researchers have also begun to view environmentalism in terms of both environmental identity [18], and environmental attitude [19], and postulate that both belief and identity held by individuals would also have important impacts on environmental intention. Dunlap et al. [19] argued that environmental attitude is a fundamental aspect of a person’s ecological worldview. An individual with an ecocentric belief places the degree of respect on the needs of the environment, while those with an anthropocentric belief view the environment as an expendable resource that is consumable for the needs of humans [19]. Clayton [18] describes environmental identity as the psychological connection between oneself and the nonhuman environment. Kashima, Paladino, and Margetts [20] argued that those with strong environmental identity are likely to show a similar tendency to regard nature as closely associated with the sphere of human activities.

Very few studies have explored the relationships between social value orientation and different aspects of environmentalism. In one recent study, Curtin and Jia [13] asked participants to write personal cooperative and competitive autobiographical memories before answering environmental questionnaires. They found that the participants who were primed with cooperative autobiographical memories scored higher in participatory environmental actions than people who were in the competitive priming group. However, priming cooperation or competition did not affect the participants’ environmental attitude or identity. These results suggest that different aspects of environmentalism should be studied in relation to social value orientation.

Although the three constructs conceptually relate to each other, the relationships among attitude, identity, and intention were complex. The authors called the attention that the link between attitude and action might be unstable. A recent study conducted by Mah, Matsuba and Pratt [21] attempted to solve the attitude-behavior gap by exploring the relationship between environmental attitude, identity, and pro-environmental action. They followed the previous findings that environmental attitude predicted connectedness/identification with nature [22]; and environmental identification positively predicted PEIs [18]. They found that environmental identity mediated the path between environmental attitude and action. These researchers argued that environmental attitude can both directly and indirectly influence PEIs via a strong identification to the nature environment (e.g., environmental identity). Thus, in the present study, we followed the previous argument that environmental attitude and identity should be the precursors of PEIs. Most importantly, we explored whether the attitude–identity–behavior path would be different in two social value orientations (prosocial and proself).

It is essential to research every possible avenue of improving the human relationship to the environment due to the severe nature of the human environmental impact. Past research did not find that social value orientations led to pro-environmental intention using the priming method. In addition, considering how broadly environmentalism had been operationalized in previous literature, a more specific approach needed to be taken. In the brief report, we first attempted to replicate the relationship by administering a decomposed game and extended the relationship to three components of environmentalism: environmental attitude, identity, and self-reported PEI. The use of several components allowed an increasingly nuanced view of how social value orientations effect environmentalism. Using the decomposed game procedure, we expected that participants in prosocial value orientation would score higher in self-reported environmental attitude, identity, and self-reported PEIs than the proself group. Secondly, we explored the attitude–identity–behavior path in the two groups (prosocial and proself value orientations) using a moderated mediation analysis. It was expected that environmental identity would mediate the environmental attitude and self-reported PEIs. The mediation relationship should be stronger in participants who were identified as prosocial value orientation than proself value orientation.

## 2. Materials and Methods

### 2.1. Participants

A priori power analysis using G*power 3.1 was conducted with two groups, three measures, within factors, alpha of 0.05. Based on a conservative guess, a small-to-moderate effect size (0.20) was estimated. The result indicated that a total sample of 132 participants was required to achieve a power of 0.95. In total, we recruited one hundred and fifty participants from the participant pool in a U.S. private university and completed an in-lab questionnaire.

Undergraduate students who were over the age of 18 and fluent in English were included and received one course credit for participation. The sample included 111 women and 38 men. One participant did not report the gender identity. The mean age of the sample was 20.53 (*SD* = 2.82) years. The majority of the participants were White (*n* = 83), followed by Hispanic/Latino (*n* = 32), Asians (*n* = 28), and African Americans (*n* = 7). The Institutional Review Board at the authors’ institution approved the project. Participants filled out an informed consent form before participating in the study.

### 2.2. Measures

Participants first completed a computer version of The Triple Dominance SVO [23]. The SVO contains a series of nine decomposed games. The decomposed game is a simplified payoff matrix with a participant believing they will be interdependently allocating resources (points) between themselves and an unknown other. The instructions did not specify the identity of the other person. Participants were told that they had never met him or her in the past and would be unlikely to meet them again in the future. The choice structure of the decomposed game is illustrated in the following: participants select A (Prosocial: maximizes joint outcome), B (Individualism: maximizes own outcome), or C (Competition: maximizes relative gain outcome). This measure has been widely used to assess social value orientations [24,25]. In order to minimize the social desirability, student research assistants administered the task. After practice trials, the participants completed the task independently without interference of the research assistants.

Following Van Lange et al.’s scoring procedure [23], each participant was only labeled in one of the SVO categories if she/he displayed at least six out of the nine decomposed games. For example, if a participant is prosocial for six games and individualism for three, the participant is placed in the prosocial value orientation category. One hundred and eight participants were categorized as prosocial; thirty-three participants were assigned to individualism; nine were assigned to competition. According to Giacomantonio et al. [4], we combined individualism and competition as proself social value orientation (*n* = 42).

We used three questionnaires to assess the environmental components of self-reported intention, identity, and attitude.

The Environmental Intention scale [26] consists of 18 items that assess a range of environmental intentions in response to the following questions: “In the last six months, how often, if at all, have you engaged in the following environmental activities and actions?” Ten items assess participatory actions (e.g., Participating in a community event which focused on environmental awareness); eight items assess leadership actions (e.g., Organizing a pro-environmental protest). Items were rated on a 5-point scale from 0 (never) through 2 (sometimes) to 4 (frequently). The Cronbach’s alpha for this scale was 0.89 (participatory subscale was 0.84; leadership subscale was 0.78) for the current study.

The Environmental Identity Scale, developed by Clayton [18], was used to measure the strength of an environmental identity. It includes 12 items rated on a Likert-type scale from 1 (not true of me at all) to 7 (completely true of me). “I feel that I receive spiritual sustenance from experiences with nature’’ is an example of an item. The Cronbach’s alpha for this scale was 0.81 for the current study.

The New Ecological Paradigm Scale by Dunlap et al. [19] is a 15-item measure that assesses environmental attitudes. Using a 9-point Likert-type scale ranging from 1 (very strongly disagree) to 9 (very strongly agree), participants rated items such as “The earth is like a spaceship with very limited room and resources” and “The balance of nature is very delicate and easily upset”. The Cronbach’s alpha for this scale was 0.80 for the current study.

After completing the decomposed game and environmental questionnaire, all participants filled out the Competitiveness Personality Scale survey [27]. It includes 9 items that are related to competitiveness. Participants rated the questionnaire on a Likert-type scale from 1 (strongly disagree) to 7 (strongly agree). A sample item of competitiveness is “I like competition because it allows me to play my best”. This measure has been used with the decomposed games to demonstrate the construct validity [27].

## 3. Results

Before testing our main hypotheses, we performed a validity check to see if the measure of decomposed game (prosocial vs. proself) was robust. According to previous studies, prosocial and proself participants scored differently regarding their competitiveness [27,28]. Analysis of Variance (ANOVA) revealed that proself participants indeed scored significantly higher in the competitiveness than the prosocial participants (*M*proself = 4.61 vs. *M*prosocial = 4.16), *F* (1, 141) = 5.14, *p* = 0.025. Thus, the validity of the decomposed game in SVO was supported.

The goal of the study was to explore the relationships between social value orientations and environmentalism. We expected that prosocial participants (who scored at least six out of the nine decomposed games on prosocial) would have higher levels of environmental identity, attitude, and intention than proself participants (who scored at least six out of the nine decomposed games on individualism and competition). To test this hypothesis, we conducted Multivariate Analysis of Covariance (MANCOVA). After controlling for gender, findings revealed significant associations between the social value orientations and three environmental variables (attitude, identity, and self-reported PEIs). *F* (3, 145) = 5.31, *p* = 0.002, *ηp*² = 0.10. The result indicated a difference in the level of social value orientations between prosocial and proself with respect to the three aspects of environmentalism. The univariate *F* tests showed there was a significant difference between prosocial and proself for environmental identity, *F* (1, 147) = 11.84, *p* = 0.001, *ηp*^2^ = 0.08, environmental attitude, *F* (1, 147) = 7.88, *p* = 0.006, *ηp*^2^ = 0.05, and environmental intention, *F* (1, 147) = 5.08, *p* = 0.026, *ηp*^2^ = 0.033. Gender was not significantly related to the environmental variables. Prosocial participants significantly scored higher than proself participants regarding the three environmental measures. In addition, we conducted a separated MANOVA in each subscale (participatory and leadership) of environmental intention. We found a significant difference between prosocial and proself participants only in participatory PEIs, *F* (1, 147) = 6.82, *p* = 0.01, *ηp*^2^ = 0.044; but not on the leadership PEIs, *F* (1, 147) = 1.24, *p* = 0.27, *ηp*^2^ = 0.008. Prosocial participants scored significantly higher than proself participants regarding the environmental participatory intentions, but not leadership. Means and Std. Errors are in Table 1.

To explore if environmental identity mediated the relationship between environmental attitude and self-reported PEIs in prosocial and proself value orientations, we conducted a moderated-mediation analysis (model 58) using the Macro PROCESS 3.1 [29]. Data were bootstrapped 5000 samples and the 95% confidence intervals were computed for the upper and lower limits of the effects. As we expected, environmental attitude positively predicted environmental identity (*b* = 0.55, *p* = 0.048); environmental identity positively predicted self-reported PEIs (*b* = 0.41, *p* = 0.01). There was no direct path between environmental attitude and self-reported PEIs (*b* = −0.04, *p* = 0.56). The index of moderated mediation index was not significant (95% CI [−0.19, 0.05]). However, we found that environmental identity mediated the relationship and the mediation path occurred in the prosocial group but not in the proself group (see Table 2). Please note that we run the same analysis regarding the two PEI subscales. The results of the moderated mediation analysis remain unchanged in participatory and leadership PEI. Please see Supplementary Materials for the additional analyses and the corresponding statistical results.

## 4. Discussion

Environmental issues are becoming increasingly prominent in today’s psychological research. Researchers have examined individual differences such as social value orientation in relation to pro-environmental intentions. Recent studies indicated priming social value orientation might not be effective to change people’s actions [14]. The present study revisited another method, namely, decomposed game to measure SVO and its relation to environmental intention. Moreover, previous studies had not directly addressed if SVO related to other environmental factors such as environmental attitude and identity. The present study addressed this gap in previous literature by comparing respective environmentalism between prosocial and proself individuals. As expected, we found that prosocial participants were more pro-environmental (including measures of self-reported pro-environmental intention, identity and attitude) than proself participants. More interestingly, we explored the environmental attitude–identity–behavior path [21]. We tested if the attitude–identity–behavior path differed in prosocial and proself groups.

Social value orientation refers to individuals’ preferences for resources allocation [1]. Two main SVOs are prosocial (the willingness to sacrifice one’s own personal gain to maximize joint gain) and proself (the desire to maximize one’s own gain relative to another individual’s personal gain) [2,4]. A well-known measure of SVOs to identifying prosocial and proself is the decomposed game [3,4,23,24,25]. Past research using the decomposed game has found that the number of prosocial and proself individuals was not balanced. For example, 27.7% of proself individuals were identified using local residents in Sweden [30]; 30% proself adults who were 35 year-old above were found in the Netherlands [31]. The results supported the current study that 27% of proself participants (*n* = 42) were identified. While we tried to minimize interference between student researchers and participants in decomposed game, we could not completely reduce the issue of social desirability. In addition, it is possible that the small percentage of the participants in proself group might reduce the predictive power. Hence, future research should consider social desirability in a rigorous methodology and increase the sample size.

Environmentalism is an abstract ideology which involves many aspects such as environmental attitude, identity, and behavior [19]. Even though these aspects are highly related to each other, conceptual and empirical differentiations have been made in the field of Environmental Psychology. For example, past research supported that environmental attitude and identity were the precursors of pro-environmental intentions and behaviors [19,20,32]. However, environmental attitude, identity, and behavior need to be disentangled. For example, Jia et al. [17] investigated values such as benevolence and generative concern (care for the future generation) in relation to environmentalism longitudinally. The authors found that early benevolence and generative concern positively predicted environmental identity and involvement, but not environmental attitude. Thus, it is important to examine different aspects of environmentalism in relation to human values.

Furthermore, environmental actions can be applied in a variety of ways. In the current study, we further examined participatory and leadership actions [26]. Accordingly, prosocial participants tended to score higher in participatory PEIs than prosocial participants; however, there was no difference in the leadership PEIs. The college sample may have a ceiling effect on the leadership PEIs. It is possible for leadership activities to require a high level of time commitment and involvement, which may be derived in the future as a result of personal interest in environmental issues [13]. Further research should investigate how different environmental actions are developed and socialized [33,34,35].

Another contribution of the present study is to explore the attitude–identity–intention path. We found that environmental attitude (NEP) did not relate to self-reported PEIs (including the two subscales). In recent studies, a weak association has also been observed between the NEP and environmental behavior [36,37]. For example, Sparks et al. [36] found that NEP for environmental attitudes did poorly (only explaining 12% of variance) in predicting pro-environmental behavior. The authors made the argument that NEP is confounded with the political orientation that may not accurately reflect the environmental attitude of ideological conservatives. In a series of psychometric tests, Sparks et al. demonstrated that other forms of environmental orientation, such as emotional connections to nature and moral environmentalism, were significantly more important in predicting environmental behavior than believing ecological paradigms as measured by NEP [37].

Moreover, similar to past research [21], environmental attitude may not necessarily lead to environmental actions. We found that environmental identity mediated the relationship between environmental attitude and self-reported PEIs. The relationship can be indirectly explained by environmental identity, a strong connection with nature and a sense of self in the natural environment. Moreover, the present study revealed that the path only existed when people were categorized as the prosocial value orientation. Future research should investigate the reason and mechanism behind this finding.

The study was not without limitations. First, the study was based on a sample of college students with an imbalanced gender distribution (over 70% were women). Although the study did not find significant gender differences on the three environmental variables, women tended to be more communal and relational than men [38]. And the decomposed game assessed communal and relational aspects of the prosocial values (e.g., considering the other person’s gain or loss). Thus, the results of the present study should not be generalized. Secondly, students may have more opportunity to be cooperative such as working in a group setting compared to when they compete against each other. Future studies should look at both social value orientation and environmentalism in a representative and diverse sample [35,39]. Thirdly, we only used the decomposed game to assess individual differences on social value orientation. Although it was a robust measure and the current study showed the construct validity, other methods such multiple players deconstructed games, prisoner’s dilemmas [24,25] and common dilemmas [40] should be incorporated. In addition, the current study only established the relationship between social value orientation and environmentalism. It did not explain the mechanism regarding why prosocial individuals were more pro-environmental than proself people, nor a causal direction. It seems also possible that individual was socialized into an eccentric worldview which, in turn, seems to have pro-social orientation. Future studies should explore other individual differences such as personality traits prosocial people possessed which potentially mediate the relationship.

## 5. Conclusions

In conclusion, we found that prosocial participants are more likely to endorse environmental attitude, identity, and PEI than proself participants. More interestingly, we found that environmental identity mediated the attitude–behavior pathway, only in a pro-social value orientation. Through an understanding of the social value orientations, it may be possible to predict pro-environmental behavior, and thus reduce the negative impact that human activities have on the environment.

## Figures and Tables

**Table 1:** Mean and Std. Errors between Groups.

	Prosocial	Proself
Environmental Identity	5.27 (0.08)	4.75 (0.13)
Environmental Attitude	5.03 (0.07)	4.66 (0.11)
Environmental Intention	2.08 (0.06)	1.85 (0.09)
Participatory Intention	2.63 (0.07)	2.29 (0.11)
Leadership Intention	1.40 (0.05)	1.30 (0.07)

**Table 2:** Moderated Mediation Model of Attitude–Identity–Behavior.

Self-Reported PEIs	*b*	*SE*	LLCI	ULCI	*t*	*p*
Constant			−1.25	1.72	0.31	0.75
Environmental Attitude	−0.04	0.06	−0.16	0.09	−0.55	0.58
Mediation in Prosocial	0.12	0.04	0.06	0.22	-	-
Mediation in Proself	0.06	0.05	−0.02	0.15	-	-

Mediation: Environmental Attitude—Environmental Identity—Self-Reported PEIs.

## Data Availability

The data presented in this study are available on request from the corresponding author. The data are not publicly available due to the policy of the Institutional Review Board.

## Supplementary Materials

The following supporting information can be downloaded at: https://www.mdpi.com/article/10.3390/ijerph19126961/s1, Moderated Mediation Analysis for Participatory and Leadership PEI.
