# Supplementary material for: Revisiting Social Value Orientations and Environmental Attitude–Identity–Intention in Decomposed Games
Source: Int J Environ Res Public Health. 2022 Jun 7;19(12):6961. doi: 10.3390/ijerph19126961 (PMC9223210; doi:10.3390/ijerph19126961)
Supplement: Supplementary file 1 [file ijerph-19-06961-s001.zip › ijerph-1746113-supplementary.pdf]

Supplementary Analyses: Moderated Mediation Analysis for Participatory and Leadership PEI

Participatory PEI: To exploring if environmental identity (mediator) mediated the relationship between *environmental attitude* and self-reported participatory PEIs; and if the environment attitude-identity-behavior path differed in prosocial and proself value orientations (moderator), we conducted a moderated-mediation analysis (model 58) using the Macro PROCESS 3.1 (Hayes et al., 2017). Data were bootstrapped 5,000 samples and the 95% confidence intervals were computed for the upper and lower limits of the effects. As we expected, environmental attitude positively predicted environmental identity (b = .55, p = .048); environmental identity positively predicted self-reported participatory PEIs (b = .57, p = .004). There was no direct path between environmental attitude and self-reported PEIs (b = -.03, p = .43). The index of moderated mediation index was not significant (95% CI [-.26, .07]. However, we found that environmental identity mediated the relationship and the mediation path occurred in the proposal group but not in the proself group.

Participatory PEIs	*b*	*SE*	LLCI	ULCI	*t*	*p*
Constant			-.06	1.02	-.06	.95
Environmental Attitude	-.03	.08	-.18	.13	-.33	.74
Mediation in Prosocial	.18	.05	.09	.30	-	-
Mediation in Proself	.08	.05	-.26	.07	-	-

Mediation: Environmental Attitude – Environmental Identity – Participatory PEIs

Leadership PEIs: We conducted the same moderated mediation analysis in leadership PEIs. We found that environmental identity did not predict self-reported leadership PEIs (b = .21, p = .14). There was no direct path between environmental attitude and self-reported PEIs (b = -.05, p = .65). The index of moderated mediation index was not significant (95% CI [-.11, .04]. Environmental identity mediated the relationship and the mediation path occurred in the proposal group (with a small effect size) but not in the proself group.

Leadership PEIs	*b*	*SE*	LLCI	ULCI	*t*	*p*
Constant			-.85	2.06	.83	.41
Environmental Attitude	-.05	.06	-.16	.06	-.90	.34
Mediation in Prosocial	.06	.03	.02	.13	-	-
Mediation in Proself	.03	.03	-.03	.10	-	-

Mediation: Environmental Attitude – Environmental Identity – Leadership PEIs
